# Menstrual cycle affects iron homeostasis and hepcidin following interval running exercise in endurance-trained women

**DOI:** 10.1007/s00421-022-05048-5

**Published:** 2022-09-21

**Authors:** Víctor M. Alfaro-Magallanes, Laura Barba-Moreno, Nuria Romero-Parra, Beatriz Rael, Pedro J. Benito, Dorine W. Swinkels, Coby M. Laarakkers, Ángel E. Díaz, Ana B. Peinado, the IronFEMME Study Group

**Affiliations:** 1 LFE Research Group, Department of Health and Human Performance. Faculty of Physical Activity and Sport Science (INEF), Universidad Politécnica de Madrid, Martín Fierro, 7, 28040 Madrid, Spain; 2 Department of Laboratory Medicine, Translational Metabolic Laboratory (TML 830), Radboud University Medical Center, P.O. Box 9101, 6500 HB Nijmegen, The Netherlands; 3 Hepcidinanalysis.Com, Geert Grooteplein 10 (830), 6525 GA Nijmegen, The Netherlands; 4 Clinical Laboratory, National Center of Sport Medicine, Health and Sports Department, AEPSAD, Madrid, Spain

**Keywords:** Anemia, Athlete monitoring, Female athletes, Inflammation, Interleukin-6, Iron deficiency

## Abstract

**Purpose:**

Menstrual cycle phase affects resting hepcidin levels, but such effects on the hepcidin response to exercise are still unclear. Thus, we investigated the hepcidin response to running during three different menstrual cycle phases.

**Methods:**

Twenty-one endurance-trained eumenorrheic women performed three identical interval running protocols during the early-follicular phase (EFP), late-follicular phase (LFP), and mid-luteal phase (MLP). The protocol consisted of 8 × 3 min bouts at 85% of the maximal aerobic speed, with 90-s recovery. Blood samples were collected pre-exercise and at 0 h, 3 h and 24 h post-exercise.

**Results:**

Data presented as mean ± SD. Ferritin were lower in the EFP than the LFP (34.82 ± 16.44 vs 40.90 ± 23.91 ng/ml, *p* = 0.003), while iron and transferrin saturation were lower during the EFP (58.04 ± 19.70 µg/dl, 14.71 ± 5.47%) compared to the LFP (88.67 ± 36.38 µg/dl, 22.22 ± 9.54%; *p* < 0.001) and the MLP (80.20 ± 42.05 µg/dl, 19.87 ± 10.37%; *p* = 0.024 and *p* = 0.045, respectively). Hepcidin was not affected by menstrual cycle (*p* = 0.052) or menstrual cycle*time interaction (*p* = 0.075). However, when comparing hepcidin at 3 h post-exercise, a moderate and meaningful effect size showed that hepcidin was higher in the LFP compared to the EFP (3.01 ± 4.16 vs 1.26 ± 1.25 nMol/l; *d* = 0.57, CI = 0.07–1.08). No effect of time on hepcidin during the EFP was found either (*p* = 0.426).

**Conclusion:**

The decrease in iron, ferritin and TSAT levels during the EFP may mislead the determination of iron status in eumenorrheic athletes. However, although the hepcidin response to exercise appears to be reduced in the EFP, it shows no clear differences between the phases of the menstrual cycle (clinicaltrials.gov: NCT04458662).

**Supplementary Information:**

The online version contains supplementary material available at 10.1007/s00421-022-05048-5.

## Introduction

Iron is an essential mineral in the functioning of the human body which is involved in several physiological processes, both at the cellular and systemic level, and whose regulation is coordinated by hepcidin (Muckenthaler et al. 2017). Hepcidin is an antimicrobial peptide synthesized in the liver that regulates iron homeostasis by binding and inducing degradation or occlusion of ferroportin, the sole known cellular iron exporter on the cell membrane (Xiao et al. 2020). This leads to reduced iron supply into plasma by blocking the absorption of dietary iron in the duodenum, iron recycling in macrophages and the release of body iron stores (Xiao et al. 2020). Iron deficiency (ID) in athletes has been shown to reduce endurance capacity and athletic performance through altered energy metabolism, decreased lactate threshold, early onset of fatigue and worsened test times (Hinton 2014; Frise et al. 2022). Furthermore, ID may negatively impact athletes' health and well-being, by affecting different domains of cognitive performance such as motivation, concentration and decision making (Pedlar et al. 2018).

Premenopausal female athletes are considered an at-risk population for ID (Pedlar et al. 2018; Sim et al. 2019). Depending on the criteria used to identify ID and the size of the study cohort, literature ranges of ID prevalence in this population from 15% to more than 50%, which contrasts with the lower prevalence range for European healthy premenopausal women (3.1–32.1%) (Milman et al. 2017; Sim et al. 2019). There are many factors that determine the regulation of iron homeostasis in athletes (Pedlar et al. 2018), which often makes maintaining a healthy iron status a challenge for female athletes and their coaches, nutritionists and clinicians. The main factors contributing to ID in both sedentary premenopausal women and premenopausal athletes are monthly hemoglobin and iron losses due to menstrual bleeding along with low dietary iron intake (Coad and Pedley 2014). On top of that, athletic populations have higher iron demands promoted by regular exercise, which is a result of higher iron losses and higher iron requirements for erythropoiesis (Pedlar et al. 2018). Furthermore, female athletes often have a poor nutritional strategy to accommodate both the additional iron demands and exercise-related energy needs (DellaValle 2013; Pedlar et al. 2018). Of note, the latter has recently been suggested to affect hepcidin regulation and may hinder iron absorption (Badenhorst et al. 2019).

Research has sought to determine the effects of exercise on iron regulation in athletes in order to determine appropriate interventions to treat and manage ID in this population. Accordingly, several studies have described the influence of endurance exercise on hepcidin regulation in both the short- and long-term (Karl et al. 2010; Auersperger et al. 2012, 2013; McClung et al. 2013; Sim et al. 2014; Ishibashi et al. 2017; Moretti et al. 2018; Larsuphrom and Latunde-Dada 2021; Hennigar et al. 2021). In the short term, exercise markedly increases hepcidin, reaching a peak at 3 h post-exercise. This peak is mediated by the release of interleukin-6 (IL-6) from contracting skeletal muscle during exercise, which peaks at the end of exercise or shortly thereafter (Pedersen and Febbraio 2008). In turn, the magnitude of the hepcidin response to exercise is also mediated by the athlete's pre-exercise iron stores, with the response being lower in those with depleted ferritin stores (Peeling et al. 2014).

To individualize and apply to athletes the most effective intervention depending on their physiology, recently the literature has gone further and described the response of hepcidin to exercise in different internal and external environments, such as under hot environments, hypoxia or in different hormonal states (Barba-Moreno et al. 2020; McKay et al. 2021; Larsuphrom and Latunde-Dada 2021; Zheng et al. 2021; Alfaro-Magallanes et al. 2021). The latter is of particular interest in the case of female athletes, as sex hormones fluctuations during the menstrual and oral contraceptive cycle seems to affect hepcidin and iron status parameters in sedentary women (Lainé et al. 2016). Indeed, the follicular phase has been postulated to offer a higher responsiveness to nutritional and supplemental iron treatment (Badenhorst et al. 2021). Therefore, pinpointing the pattern of hepcidin and iron markers throughout the menstrual cycle would be useful for determining the iron status of eumenorrheic athletes, as well as for the potential identification of low energy availability in this population (Badenhorst et al. 2019). Two studies have investigated the acute response of hepcidin to exercise (0 and 3 h post-exercise) in different phases of the menstrual cycle, observing an absence of significant differences between menstrual cycle phases (Barba-Moreno et al. 2020; Zheng et al. 2021).

It should be noted that these studies used different methodologies to determine the phases of the menstrual cycle and different hormonal phases to be analyzed within the menstrual cycle, which makes them difficult to compare. To solve the issue of the use of different methodologies that frequently occurs in women's studies, working guidelines for standards of practice for research on women have recently emerged. These guidelines facilitate the study of the phases of the menstrual cycle with the most pronounced hormonal changes and the obtaining of valid and comparable results between studies (Schaumberg et al. 2017; Janse de Jonge et al. 2019; Elliott-Sale et al. 2021). However, Barba-Moreno et al.(2020) and Zheng et al. (2021) did not conform to this recent methodology for the study of the hepcidin response to exercise across the menstrual cycle, either because they did not use the three-step method to verify the menstrual cycle (Barba-Moreno et al. 2020) or because they did not select the three phases of the menstrual cycle with the most pronounced hormonal changes (Barba-Moreno et al. 2020; Zheng et al. 2021). Complying with these guidelines will ensure valid results and may aid the correct diagnosis of ID in females with respect to the distinct phases of the menstrual cycle. Therefore, the aim of this study was to investigate the hepcidin response to interval running exercise during the three phases of the menstrual cycle with the most pronounced hormonal changes by carrying out the three-step method for menstrual cycle verification (Elliott-Sale et al. 2021).

## Methods

### Participants

Twenty-one healthy, endurance-trained females with eumenorrheic cycles [age: 30.5 ± 6.5 years; height: 1.63 ± 0.06 m; body mass: 58.4 ± 8.7 kg; body fat: 26.4 ± 7.0%; peak oxygen consumption (${\dot{\text{V}}\text{O}}_{{{\text{2peak}}}}$): 48.4 ± 4.4 mL min^–1^ kg^–1^] were recruited for this study. Training status of the participants was considered as trained/developmental according to the training status classification proposed by McKay et al. (2022). An eumenorrheic menstrual cycle was defined as a regularly occurring menstrual cycle ranging from 24 to 35 days in length (Lebrun et al. 1995; Middleton and Wenger 2006). Participants possessed 7.4 ± 5.3 years of endurance training experience, with a training volume of 296 ± 184 min per week during the 6 months prior to recruitment. The calculation of the sample size suggested 21 participants to produce a statistical power of 0.80 with an effect size of 0.75 at a significance level of *p* < 0.05 (see calculation details here (Peinado et al. 2021)). To be included in the study, participants were required to meet the following criteria: (i) healthy adult females between 18 and 40 years of age; (ii) naturally menstruating for the 6 months prior to the study, and during the study, to present with eumenorrheic cycles confirmed by peak luteinizing hormone (LH) and sex hormone concentrations; (iii) no consumption of medication that alters vascular function (e.g., tricyclic antidepressants, α-blockers, β-blockers, etc.) or any dietary supplements (including any iron supplementation); (iv) non-smokers; (v) non-pregnant or oophorectomized; (vi) participates in endurance training between 3 and 12 h per week; and (vii) no IDA (serum ferritin: < 20 ng/ml; hemoglobin: < 11.5 g/dl; and transferrin saturation: < 16% (Peeling et al. 2007)). To verify inclusion criteria, a self-reported questionnaire and a blood analysis were provided by the participants (Peinado et al. 2021). The Research Ethics Committee of the Universidad Politécnica de Madrid approved the project, and participants provided written informed consent. This study is registered on clinicaltrials.gov (ID: NCT04458662).

### Menstrual cycle monitoring

Participants performed the main project protocol during the following menstrual cycle phases: early-follicular phase (EFP), late-follicular phase (LFP), and mid-luteal phase (MLP). Each participant’s menstrual cycle length was calculated by a gynecologist using the calendar-based method with the information of the last six menstrual cycles prior to joining the study (Janse de Jonge et al. 2019). Participants were scheduled on the dates indicated for the above menstrual cycle phases. EFP was determined by the onset of menses. The LFP was scheduled between 1 and 3 days before expected ovulation (mid-cycle), and the MLP was between 5 and 9 days after confirmed ovulation. Ovulation was confirmed using at home urinary ovulation tests (Ellatest, Alicante, Spain). For the use of these tests, the second morning mid-stream urine sample was collected every day from 3 to 5 days before the LFP protocol until LH surge detection. This surge allegedly occurs 14–26 h before ovulation (Janse de Jonge et al. 2019). If a LH surge was not detected or was detected more than 3 days after completion of the LFP test, the test was discarded and the dates for the LFP test were recalculated to repeat the testing session. Lastly, a blood sample was collected before every trial to analyze sex hormones and retrospectively confirm the phase of the menstrual cycle. No participant presented with a deficient luteal phase, considered as such when progesterone is below 5.03 ng/ml (Janse de Jonge et al. 2019; Elliott-Sale et al. 2021).

### Study design

Participants were required in the laboratory four times over a three-month period. On the first occasion, they underwent a screening protocol during the EFP since the lowest levels of iron-related parameters have been reported during this phase (Lainé et al. 2016). Firstly, baseline blood samples were collected in a rested and fasted state. A complete blood count and biochemistry and hormonal analysis were performed to verify the conformity of iron-metabolism-related parameters with the inclusion criteria and to dismiss any illness, hormonal disorders, or menstrual cycle dysfunction. On the same day, after an adequate meal and rest (a minimum of 2 h after feeding), participants performed a maximal ramp test on a computerized treadmill (H/P/COSMOS 3PW 4.0; H/P/Cosmos Sports & Medical GmbH, Nussdorf-Traunstein, Germany) to determine each participant’s ${\dot{\text{V}}\text{O}}_{{{\text{2peak}}}}$ and maximal aerobic speed (${{s\dot{V}O}}_{{{\text{2peak}}}}$), as previously described (Peinado et al. 2021).

**Fig. 1:**
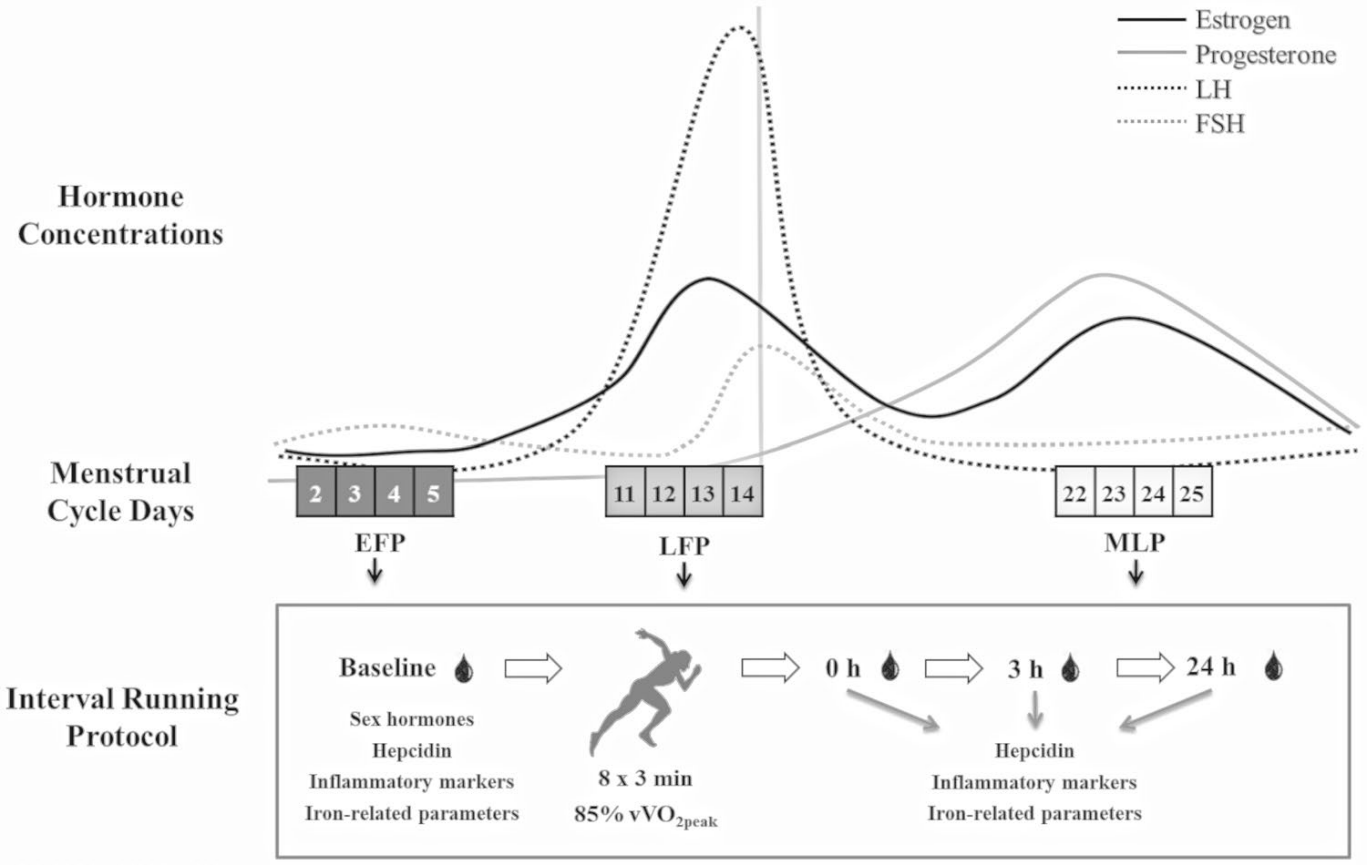
Experimental protocol performed in the EFP, LFP, and MLP of the menstrual cycle. *EFP* early-follicular phase, *FSH* follicle-stimulating hormone, *LFP* late-follicular phase, *LH* luteinizing hormone, *MLP* mid-luteal phase, 0 h, 0 h post-exercise, 3 h, 3 h post-exercise, 24 h, 24 h post-exercise

After this screening day, participants reported to the laboratory for the interval running protocol during the mentioned menstrual cycle phases: EFP (day 3.4 ± 0.9), LFP (day 12.0 ± 2.5), and MLP (day 21.9 ± 3.1) (see Fig. 1). In addition, the mean day of the positive result in the LH test was 14.0 ± 2.6. The order of these running protocols was randomized and counterbalanced in order to avoid a learning effect in our participants, which could affect our results. The following test orders were randomized, and in no case, an order involved evaluating a volunteer in more than two menstrual cycles: EFP-LFP-MLP, LFP-MLP-EFP, MLP-EFP-LFP, LFP-EFP-MLP, and EFP-MLP-LFP.

### Interval running protocol

Participants abstained from alcohol, caffeine, and any intense physical activity or sport from 24 h before coming to the laboratory until the last blood collection was done (24 h post-exercise). Protocols were initiated between 8 and 10 AM to avoid diurnal variability of hepcidin (Troutt et al. 2012) and after the participants had breakfast at least 2 h earlier. Nutritional recommendations were provided to all participants by a nutritionist in order to avoid potential pro-inflammatory effects, such as those caused by red and processed meat (Chai et al. 2017), and to prevent dietary supplements or dietary plan (Pasiakos et al. 2016) from masking inflammatory markers and hepcidin results. These recommendations were followed by all participants from 48 h before the exercise protocol to 24 h after completing it. Firstly, a blood sample was collected before the running protocol to analyze sex hormones and parameters related with iron homeostasis. Subsequently, participants started the interval running protocol. This consisted of a 5-min warm-up at 60% of the ${{s\dot{V}O}}_{{{\text{2peak}}}} {,}$ followed by 8 bouts of 3 min at 85% of the ${{s\dot{V}O}}_{{{\text{2peak}}}}$ with a 90-s recovery at 30% of the ${{s\dot{V}O}}_{{{\text{2peak}}}}$ between bouts, followed by a final 5-min cooling down period at 30% of the ${{s\dot{V}O}}_{{{\text{2peak}}}}$. This protocol was previously reported by Sim et al. (2013) to produce enough of a stimulus to cause an increase in hepcidin 3 h post-exercise. In addition to the pre-exercise sample, antecubital venous blood samples were collected on 3 different occasions post-exercise: just after finishing the interval protocol (0 h), 3 h, and 24 h after finishing the interval protocol. Participants were allowed to drink water ad libitum between 0 and 3 h post-exercise, but no food was consumed. After 3 h, they followed the aforementioned dietary recommendations until the 24 h post-exercise blood collection. The participants maintained a food diary starting 48 h before the running protocol and lasting 24 h after the protocol (see Supplementary table 1 for nutritional intake in each menstrual cycle phase).

### Blood collection and analysis

IL-6, tumor necrosis factor alpha (TNF-α), iron, ferritin, transferrin, and C-reactive protein (CRP) were analyzed in all samples at the clinical laboratory of the Spanish National Centre of Sport Medicine (Madrid, Spain). Inflammatory markers (IL-6, TNF-α and CRP) were measured to screen for any effect of inflammation on hepcidin and iron markers, and to verify that the increase in IL-6 produced after the protocol was mainly muscle-derived and not also due to an inflammatory response to exercise (Pedersen and Febbraio 2008; Schmidt 2015). Additionally, pre-exercise serum samples were also analyzed for 17 β-Estradiol, progesterone, LH, follicle-stimulating hormone (FSH), and prolactin. Transferrin saturation (TSAT) was calculated from iron and transferrin measurements as previously indicated (Eleftheriadis et al. 2010). The handling and processing of the blood samples, as well as the methods of analysis and coefficients of variation reported by the laboratory for each of the mentioned variables can be found in Peinado et al. (2021).

Duplicate serum samples were sent to the Department of Laboratory Medicine at Radboud University Medical Centre (Hepcidinanalysis.com, Nijmegen, The Netherlands) for measurement of hepcidin-25 serum concentrations. Hepcidin measurements were performed in June 2019 by a weak cation exchange chromatography followed by time-of-flight mass spectrometry (WCX-TOF MS) using a stable hepcidin-25 + 40 isotope as internal standard for quantification (Laarakkers et al. 2013). Peptide spectra were generated on a Microflex LT matrix-enhanced laser desorption/ionization TOF MS platform (Bruker Daltonics). The assay is standardized using secondary reference material for hepcidin assays. To allow traceability to the internationally recognized Système International of this secondary reference material, its value is assigned from a primary reference material (Diepeveen et al. 2019; Aune et al. 2020). Hepcidin-25 concentrations were expressed as nMol/L. The lower limit of quantification of this method was 0.5 nMol/L. Inter-assay CV for hepcidin was 4.6% at the 11.0 nMol/L level and 8.3% at the 2.7 nMol/L level. Reference values for hepcidin and its ratio to TSAT and ferritin can be found at the website of Hepcidinanalysis.com (accessed on January 13th, 2021).

### Statistical analysis

Data are presented as the mean and standard error of the mean (± SEM). A Shapiro–Wilk test was conducted to assess the normality of all the variables. A linear mixed model for repeated measures was used to analyze hepcidin, IL-6, TNF-α, ferritin, iron, transferrin, TSAT and CRP. For this analysis, the “menstrual cycle phase” (EFP, LFP, and MLP) and the “time of measurement” (pre-exercise, 0 h post-exercise, 3 h post-exercise, and 24 h post-exercise) were set as fixed factors. The interaction of these two factors (menstrual cycle*time of measurement) was analyzed to investigate the response of the above variables to exercise in the different menstrual cycle phases. In addition, to account for natural within-participant variability in the measured physiological variables, subjects were included as a random factor ("subject") in the linear mixed model. A Bonferroni post-hoc test was conducted where significant differences were found in any of the analyzed fixed factors. A non-parametric Friedman ANOVA for repeated measures was performed to analyze differences in sex hormone concentrations between the menstrual cycle phases tested. A non-parametric Wilcoxon signed-rank test was performed to obtain post-hoc pairwise comparisons where significant differences were found. Effect sizes for non-parametric pairwise comparisons were calculated using coefficient r (Rosenthal 1991), while for Bonferroni post-hoc comparisons, Cohen’s d (Cohen 1988) was calculated to assess the magnitude of the effect on the changes found. To unify the effect size under a sole coefficient, *r* values were converted to d values, as proposed by Rosenthal (Rosenthal 1991). Threshold values were set as small (≥ 0.2 and < 0.5), moderate (≥ 0.5 and < 0.8), and large (≥ 0.8) (Cohen 1988). In addition, 95% confidence intervals (CI) were calculated. The statistical significance was set at *p* < 0.05. In addition, the effect size was considered meaningful when its CI did not include zero (Nakagawa and Cuthill 2007). All procedures were conducted with SPSS software, version 25 (IBM Corp., Armonk, NY, USA).

## Results

### Sex hormones

Sex hormone concentrations between menstrual cycle phases tested (see Table 1) showed significant differences for 17 β-Estradiol (χ^2^ = 25.810; *p* < 0.001), progesterone (χ^2^ = 25.810; *p* < 0.001), LH (χ^2^ = 9.810; *p* = 0.007), and FSH (χ^2^ = 30.095; p < 0.001). 17 β-Estradiol exhibited lower values during the EFP in comparison with the LFP (*p* < 0.001, *d* = 1.79, CI = 0.99–2.59) and MLP (p < 0.001, d = 1.91, CI = 1.16–2.66), while progesterone showed higher concentrations during the MLP than in the EFP (*p* < 0.001, *d* = 1.79, CI = 0.97–2.61) and LFP (*p* < 0.001, *d* = 1.91, CI = 1.08–2.74). In addition, LH levels were higher during the LFP in comparison with the MLP (*p* = 0.006, *d* = 1.08, CI = 0.41–1.75). This was coupled with lower FSH concentrations during the MLP than in the EFP (*p* < 0.001, *d* = 2.76, CI = 1.83–3.69) and LFP (*p* < 0.001, *d* = 1.58, CI = 0.89–2.27) Table 2.

**Table 1:** Resting serum concentrations of sex hormones presented as mean ± SD

		EFP	LFP	MLP
17 β-Estradiol (pg/ml)		38.78 ± 30.39	186.67 ± 156.27^Ω^	138.11 ± 71.99^Ω^
Progesterone (ng/ml)		0.33 ± 0.19	0.75 ± 1.79	12.00 ± 5.37^Ω£^
LH (mIU/ml)		7.27 ± 3.91	12.56 ± 7.36	5.96 ± 3.26
FSH (mIU/ml)		9.14 ± 8.49	6.17 ± 2.72	3.44 ± 1.53^Ω£^
Prolactin (mIU/l)		483.58 ± 302.63	415.03 ± 149.09	514.47 ± 229.30

*EFP* early-follicular phase, *FSH* follicle-stimulating hormone, *LFP* late-follicular phase, *LH* luteinizing hormone, *MLP* mid-luteal phase. Ω Significantly different from EFP. £ Significantly different from LFP

**Table 2:** Hepcidin, inflammatory markers, and iron-related parameters throughout the menstrual cycle presented as mean ± SD

	EFP	LFP	MLP
Hepcidin (nMol/l)	1.02 ± 1.37	1.81 ± 2.86	1.24 ± 1.46
Interleukin-6 (pg/ml)	2.54 ± 1.76	2.48 ± 2.03	2.22 ± 1.47
TNF-⍺ (pg/ml)	4.96 ± 1.27	4.95 ± 1.75	4.68 ± 1.21
CRP (mg/l)	1.23 ± 1.04	0.65 ± 0.48^Ω^	0.80 ± 0.65^Ω^
Iron (µg/dl)	58.04 ± 19.70	88.67 ± 36.38^Ω^	80.20 ± 42.05^Ω^
Ferritin (ng/ml)	34.82 ± 16.44	40.90 ± 23.91^Ω^	37.37 ± 22.89
Transferrin (mg/dl)	286.04 ± 34.53	288.48 ± 33.84	290.57 ± 33.57
TSAT (%)	14.71 ± 5.47	22.22 ± 9.54^Ω^	19.87 ± 10.37^Ω^

*CRP* C-reactive protein, *EFP* early-follicular phase, *LFP* late-follicular phase, *MLP* mid-luteal phase, *TNF-⍺* tumor necrosis factor alpha, *TSAT* transferrin saturation. Ω Significantly different from EFP

### Hepcidin

**Fig. 2:**
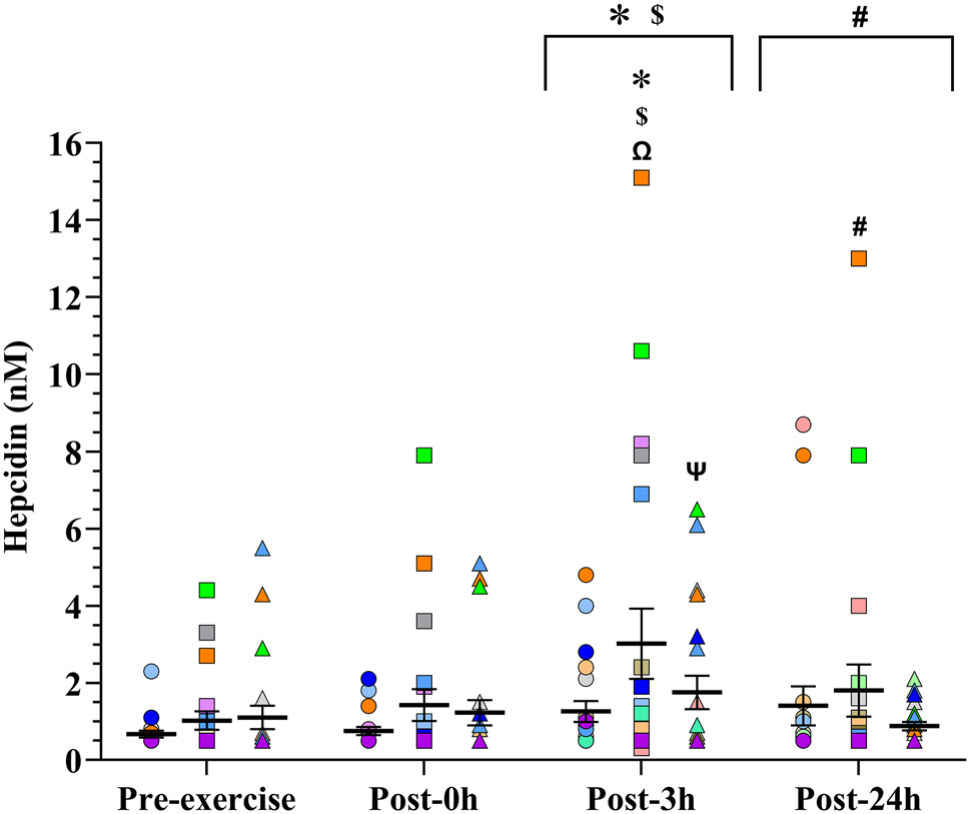
Mean (SEM) serum hepcidin concentrations and the individual hepcidin response of each participant to the interval running protocol in the early-follicular phase (circles), late-follicular phase (squares) and mid-luteal phase (triangles). Each color represents a different participant. Symbols above the arrows indicate post-hoc differences for the factor Time. Post-0 h, 0 h post-exercise; Post-3 h, 3 h post-exercise; Post-24 h, 24 h post-exercise *Significantly different from Pre-exercise. $ Significantly different from Post-0 h. # Significantly different from Post-3 h

The hepcidin response to exercise showed a significant effect of time (*F*_3.180_ = 7.179, *p* < 0.001) with higher concentrations at 3 h post-exercise than pre-exercise (*p* < 0.001, *d* = 0.51, CI = 0.16–0.86), 0 h (*p* = 0.001, *d* = 0.39, CI = 0.13–0.65), and 24 h post-exercise (*p* = 0.026, *d* = 0.25, CI =  – 0.14 to 0.64) (post-hoc differences are showed in Fig. 2, for mean ± SEM of time effects see Supplementary Table 2). Statistical analysis showed no effect of the menstrual cycle phase on hepcidin concentrations (*F*_2.50_ = 3.131; *p* = 0.052). Similarly, our results presented no significant effects of the interaction between time and the menstrual cycle phase (*F*_6.167_ = 1.955; *p* = 0.075). However, meaningful effect sizes were found for some specific time points in different menstrual cycle phases. Specifically, focusing on the hepcidin response at 3 h post-exercise, a moderate and meaningful effect size was found between the LFP and the EFP (*d* = 0.57, CI = 0.07–1.08). Looking at pre-exercise hepcidin concentrations, a small but meaningful effect size was found, suggesting higher hepcidin levels at pre-exercise during the MLP in comparison to the EFP (*d* = 0.42, CI = 0.06–0.78). Lastly, the LFP was the only phase showing a clear hepcidin response to the exercise protocol (*F*_3.173_ = 7.902; *p* < 0.001), with higher hepcidin levels at 3 h post-exercise than pre-exercise (*p* < 0.001, *d* = 0.66, CI = 0.34–0.97), 0 h (*p* < 0.001, *d* = 0.49, CI = 0.23–0.76), and 24 h post-exercise (*p* = 0.014, *d* = 0.33, CI = 0.004–0.66). However, the hepcidin response to the exercise protocol was not significant during the EFP (*F*_3.173_ = 0.933; *p* = 0.426) and MLP (*F*_3.173_ = 1.865; *p* = 0.137). The individual hepcidin response of each participant at each phase of the menstrual cycle is showed in Fig. 2.

### Inflammatory markers

**Fig. 3:**
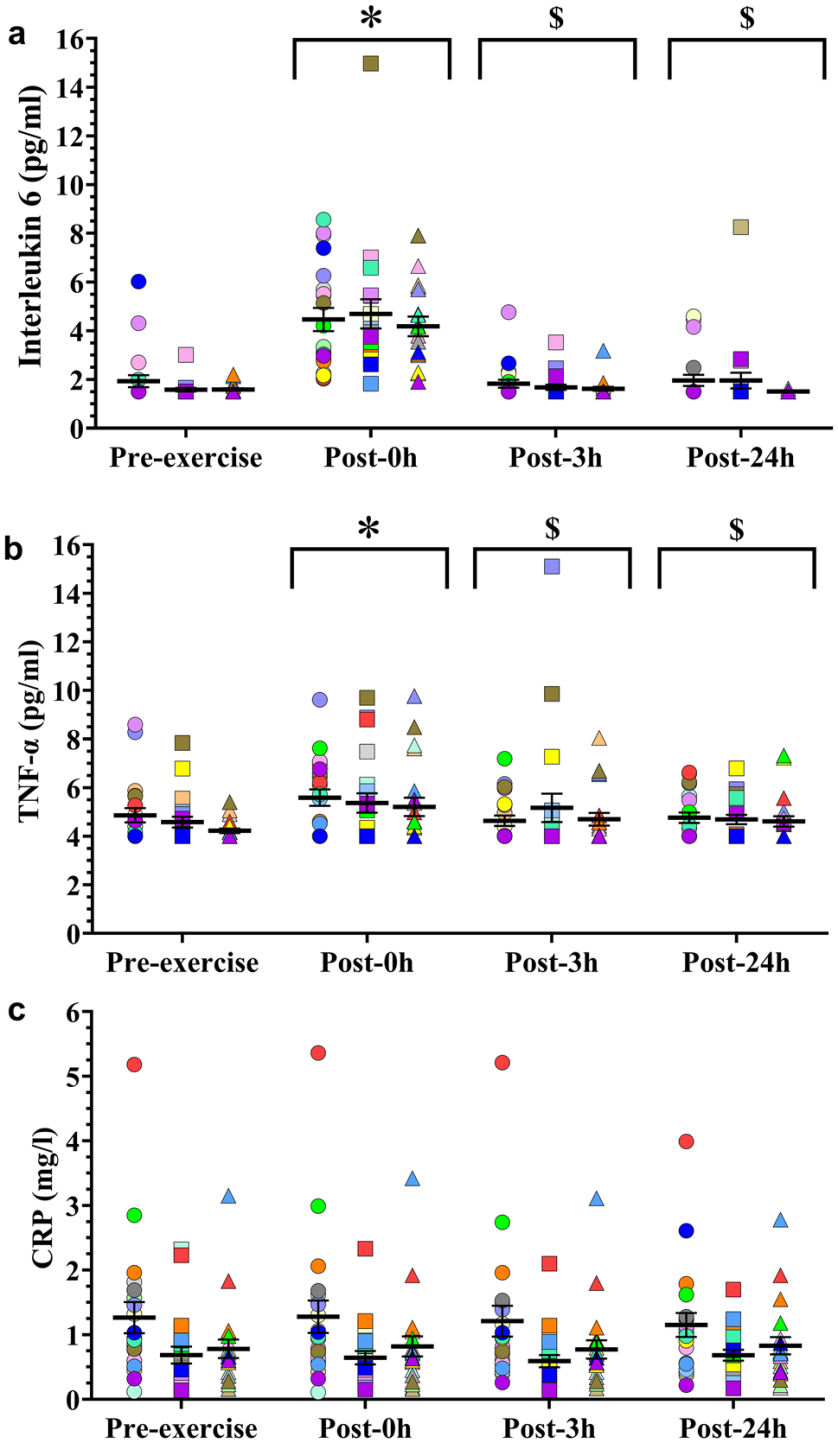
Mean (SEM) serum IL-6 (**a**), TNF-⍺ (**b**) and CRP (**c**) concentrations and each participant's individual response to the interval running protocol in the early-follicular phase (circles), late-follicular phase (squares) and mid-luteal phase (triangles). Each color represents a different participant. Symbols above the arrows indicate post-hoc differences for the factor Time. *CRP* C-reactive protein, Post-0 h, 0 h post-exercise, Post-3 h, 3 h post-exercise, Post-24 h, 24 h post-exercise, *TNF-⍺* tumor necrosis factor alpha. Symbols above the arrows indicate post-hoc differences for the factor Time. *Significantly different from Pre-exercise. $ Significantly different from Post-0 h

Time effects were recorded for IL-6 (*F*_3.172_ = 77.208, *p* < 0.001) and TNF- α (*F*_3.168_ = 7.27; *p* < 0.001), but not for CRP (*F*_3.160_ = 0.506; *p* = 0.679) (post-hoc differences are showed in Fig. 3, for mean ± SEM of time effects see Supplementary Table 2). IL-6 presented higher levels at 0 h post-exercise compared to pre-exercise (*p* < 0.001, *d* = 1.64, CI = 0.93–2.34), 3 h (*p* < 0.001, *d* = 1.66, CI = 0.97–2.36), and 24 h post-exercise (*p* < 0.001, *d* = 1.49, CI = 0.78–2.20). TNF-α exhibited behavior similar to IL-6 with higher levels at 0 h post-exercise in comparison with pre-exercise (*p* < 0.001, *d* = 0.60, CI = 0.11–1.08), 3 h (*p* = 0.024, *d* = 0.32, CI =  – 0.08 to 0.72), and 24 h post-exercise (*p* = 0.003, *d* = 0.49, CI =  – 0.04 to 1.03).

IL-6 (*F*_2.85_ = 1.29; *p* = 0.280) and TNF-α (*F*_2.80_ = 1.432; *p* = 0.245) were not affected by the menstrual cycle phase. In contrast, CRP showed significant differences between phases (*F*_2.69_ = 11.615; *p* < 0.001), being higher during the EFP compared to the LFP (*p* = 0.001, *d* = 0.71, CI = 0.31–1.12) and MLP (*p* = 0.047, *d* = 0.49, CI =  – 0.04 to 1.03).

No significant differences were found for the interaction between time and the menstrual cycle phase for IL-6 (*F*_6.170_ = 0.343; *p* = 0.913), TNF-α (*F*_6.166_ = 0.755; *p* = 0.606) and CRP (*F*_6.197_ = 0.335; *p* = 0.918). The individual response of the inflammatory markers for each participant at each menstrual cycle phase is showed in Fig. 3.

### Iron-related parameters

**Fig. 4:**
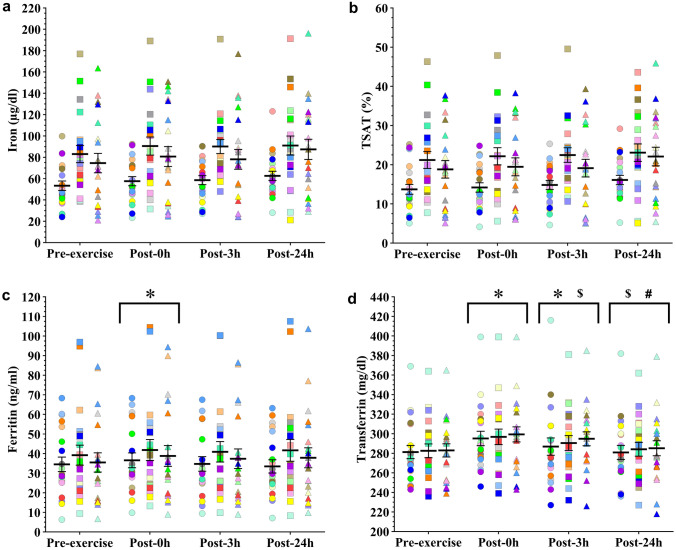
Mean (SEM) serum iron (**a**), TSAT (**b**), ferritin (**c**) and transferrin (**d**) concentrations and each participant's individual response to the interval running protocol in the early-follicular phase (circles), late-follicular phase (squares) and mid-luteal phase (triangles). Each color represents a different participant. Symbols above the arrows indicate post-hoc differences for the factor Time. Post-0 h, 0 h post-exercise, Post-3 h, 3 h post-exercise, Post-24 h, 24 h post-exercise, *TSAT* transferrin saturation. Symbols above the arrows indicate post-hoc differences for the factor Time. *Significantly different from Pre-exercise. $ Significantly different from Post-0 h. # Significantly different from Post-3 h

Time effects were observed for ferritin (*F*_3.181_ = 4.161; *p* = 0.007) and transferrin (*F*_3.17_ = 28.624; *p* < 0.001), while iron (*F*_3.187_ = 2.509; *p* = 0.060) and TSAT (*F*_3.187_ = 2.432; *p* = 0.066) did not present any differences. Post-hoc comparisons showed that ferritin concentration was higher at 0 h post-exercise compared with pre-exercise (*p* = 0.006, *d* = 0.12, CI = 0.06–0.18), while transferrin presented multiple post-hoc interactions: 0 h post-exercise transferrin levels were higher than pre-exercise (*p* < 0.001, *d* = 0.45, CI = 0.24–0.66), 3 h (*p* = 0.002, *d* = 0.18, CI = 0.03–0.33), and 24 h post-exercise levels (*p* < 0.001, *d* = 0.41, CI = 0.19–0.62). Moreover, transferrin was also higher 3 h post-exercise than pre-exercise (*p* < 0.001, *d* = 0.25, CI = 0.08–0.43) and 24 h post-exercise (*p* < 0.001, *d* = 0.21, CI = 0.04–0.39) (post-hoc differences are showed in Fig. 4, for mean ± SEM of time effects see Supplementary Table 2).

In addition, serum iron (*F*_2.53_ = 10.915; *p* < 0.001), TSAT (*F*_2.53_ = 10.4; *p *< 0.001) and ferritin (*F*_2.55_ = 6.908; *p* = 0.002) concentrations were affected by the menstrual cycle phase, unlike transferrin. Specifically, ferritin levels presented lower values in the EFP than in the LFP (*p* = 0.003, *d* = 0.30, CI = 0.03–0.56). This was coupled with lower serum iron during the EFP in comparison with the LFP (*p* < 0.001, *d* = 1.05, CI = 0.37–1.72) and MLP (*p* = 0.024, *d* = 0.68, CI = 0.08–1.27), along with lower TSAT during the EFP in comparison with the LFP (*p* < 0.001, *d* = 0.97, CI = 0.38–1.55) and MLP (*p* = 0.045, *d* = 0.62, CI = 0.02–1.23).

Lastly, no significant effects were found for the interaction between time and the menstrual cycle phase for ferritin (*F*_6.189_ = 0.324; *p* = 0.924), iron (*F*_6.179_ = 0.193; *p* = 0.979), TSAT (*F*_6.181_ = 0.244; *p* = 0.961), and transferrin (*F*_6.164_ = 0.346; *p* = 0.911). The individual response of the iron-related parameters for each participant at each menstrual cycle phase is showed in Fig. 4.

## Discussion

To our knowledge, we show for the first time differences in the regulation of hepcidin along with changes in iron, TSAT and ferritin throughout the early follicular, late follicular and mid-luteal phases of the menstrual cycle in women who performed running exercise. Specifically, iron markers showed lower concentrations during the EFP, while hepcidin and its response to exercise also appeared to be slightly lower.

As it has been consistently stated in the literature (Domínguez et al. 2018; Larsuphrom and Latunde-Dada 2021), we observed a significant increase in IL-6 at 0 h post-exercise with a consequent rise in hepcidin concentration at 3 h post-exercise both compared to pre-exercise levels. Interestingly, the overall hepcidin response to the protocol trended to be higher during the LFP than the EFP, supported by a moderate and significant effect size. Firstly, it should be noted that of the two main determinants of the hepcidin response to exercise (IL-6 levels and iron status (Domínguez et al. 2018)), only iron status showed changes across the menstrual cycle. It is worth mentioning that CRP showed higher levels during the EFP, which is consistent with an inverse relationship between serum estradiol and CRP in premenopausal women (Park and Lee 2020). However, although a marker of inflammation, CRP does not activate any of the known hepcidin regulatory pathways (Xiao et al. 2020) and the magnitude of the changes found are too small to be reflected in iron markers (Galetti et al. 2021). Therefore, apart from a potential direct effect of sex hormones on the hepcidin signaling pathway in hepatocytes (Xiao et al. 2020), it seems that systemic hepcidin changes across the menstrual cycle are mainly regulated by the fluctuations found for iron, TSAT and ferritin.

Menstrual blood loss is noted as the main cause of a reduction in iron, TSAT and ferritin concentrations during the EFP (Pedlar et al. 2018; Sim et al. 2019), as found in our participants. Some days later (~ 8) in the LFP, they exhibited improved serum ferritin, serum iron and TSAT. Presumably, the absence of iron loss through menstrual bleeding after the EFP phase and the low resting serum hepcidin concentrations observed in our participants allowed this improvement in iron markers. It should be noted that our participants had low resting hepcidin concentrations (see pre-exercise values in Fig. 2) compared to baseline values for sedentary premenopausal women (from 2.6 to 4.8 nMol/L) (Galesloot et al. 2011), which may be indicative of increased iron uptake in athletic women to respond not only to the demands that the menstrual cycle places on iron homeostasis, but also to those of exercise. Interestingly, iron status did not improve in the LFP compared to the MLP. The cessation of improvement in iron parameters may correspond to a stabilization of hepcidin levels during the luteal phase, as proposed in sedentary premenopausal women (Lainé et al. 2016). Although not statistically significant, the effect sizes found between our pre-exercise hepcidin values align with this proposal. In turn, the observed improvements in iron markers in LFP compared to EFP may be a reason by itself, at least partially, for hepcidin stabilization and, consequently, not finding more improvements in iron status in the MLP compared to the LFP. In addition, sex hormones may also have played a role, as progesterone and estrogen have been shown to be able to modulate hepcidin independently of the BMP-SMAD signaling pathway in animal studies (Hou et al. 2012; Yang et al. 2012; Li et al. 2015). However, it would be premature to conclude that the reason for the stabilization of hepcidin and iron parameters is simply due to a direct effect of sex hormones.

Previous studies investigating iron homeostasis within the menstrual cycle also found reduced serum iron, ferritin, and/or TSAT during the EFP compared with other phases of the menstrual cycle (Kim et al. 1993; Lainé et al. 2016; Barba-Moreno et al. 2020). However, most of them did not carry out menstrual cycle phase verification and there also exist conflicting results (Puolakka 1980; Heath et al. 2001; Belza et al. 2005; Zheng et al. 2021). Similar to our results, Lainé et al. (2016) reported changes in resting hepcidin concentrations over the menstrual cycle, which decreased during the EFP, increased during the middle of the cycle (LFP), and stabilized during the second part of the cycle (MLP). However, 54 of the 90 healthy women in the study by Lainé et al. (2016) were using oral contraceptives, which confounds results by having different hormonal profiles within the study sample. As previously discussed, the major challenge in comparing these studies is the lack of consistency between methodologies around the menstrual cycle, which hinders the development of accurate conclusions (Elliott-Sale et al. 2021).

The hepcidin response at 3 h after exercise, although not statistically significant, was moderately greater in the LFP than in the EFP, as indicated by the meaningful effect size found. However, it is also true that, although the hepcidin responses to exercise are subject to inevitable within-subject random variation (Atkinson et al. 2019), the response in the LFP appears to be highly variable in our participants compared to the EFP (see Fig. 2). This is probably because of the clear improvement that participants experienced in their iron markers during the LFP. Fluctuations in iron markers probably account for part of the lack of differences found in hepcidin response to exercise within the EFP and MLP, especially during the EFP in which iron markers levels are severely reduced. In this regard, it seems reasonable that, in response to iron loss through menstruation, the hepcidin response may be appropriately adjusted by showing a concomitant reduction in its magnitude to maximize iron absorption. This has already been demonstrated in iron-depleted individuals (Peeling et al. 2014) and, indeed, some participants became iron-depleted during the EFP due to the aforementioned iron losses. This suggests that it is not the effects of sex hormones on the hepcidin signaling cascade that govern the hepcidin response to exercise in women, but rather changes in iron homeostasis that modulate this response. The overall hepcidin response to the same exercise protocol in the different phases of the menstrual cycle for the same woman would probably be of a similar magnitude if these phases did not modulate iron homeostasis through different processes, some of them known, but others not yet (Badenhorst et al. 2021). Currently, studies with robust methodologies around the menstrual cycle are lacking to corroborate our findings, however results from Barba-Moreno et al. (2020), and Zheng et al. (2021) are mostly consistent with this theory.

Despite the aforementioned constraints, the decline of iron markers during EFP is a common occurrence (Kim et al. 1993; Lainé et al. 2016; Barba-Moreno et al. 2020; Badenhorst et al. 2021), which in addition to the large reduction in serum iron and TSAT found in our participants during the EFP, suggest important implications. Firstly, the diagnosis of ID in endurance-trained eumenorrheic women may be confounded due to the phase of the menstrual cycle in which the blood sample is taken, especially in women with iron markers bordering on ID, so these variations should be considered from a clinical standpoint. Considering the phase of the menstrual cycle at the time of sampling would help to be more accurate in the diagnosis of ID in female athletes, likely narrowing the wide range of prevalence shown by the literature (from 15% to more than 50%) (Sim et al. 2019). Secondly, given the low TSAT found during the EFP, iron supply for erythropoiesis may be compromised during this phase, resulting in iron-deficient erythropoiesis (Peeling et al. 2007). This may temporarily limit hematological adaptations to training and/or compliance with red blood cell turnover but this remains to be investigated. Female athletes appear to have a lower overall hepcidin response to exercise in the EFP, which suggests that iron absorption from both diet and supplementation may be greater at this time (Badenhorst et al. 2021). Therefore, increasing iron intake during the EFP may be a reasonable strategy to ensure adequate iron supply, not only in ID and IDA endurance-trained athletes, but also in those that present as borderline ID (serum ferritin ~ 30–35 ng/ml).

The present study takes into consideration the main hormonal environments occurring throughout the menstrual cycle, with a robust methodology to identify them, as recently suggested in the working guidelines for standards of practice for women's research (Janse de Jonge et al. 2019; Elliott-Sale et al. 2021). It should be noted that the selected running protocol might be a typical day-to-day workout for these women. Furthermore, excluding women with IDA from the study rather than women with depleted ferritin stores makes our study sample more likely to resemble the population of physically active women (Pedlar et al. 2018; Sim et al. 2019). However, as discussed, the fact that not all our participants presented replete ferritin stores affects the hepcidin response to exercise (Peeling et al. 2014). Indeed, although all participants had ferritin stores above 20 ng/ml at the screening protocol, some of them experienced a reduction in their ferritin levels below this cutoff point during the study period. This probably contributed to the limited hepcidin response to the exercise protocol. It is also worth mentioning that the mean resting hepcidin concentrations of our participants was below 1 nMol/L, which suggests increased iron absorption (Galetti et al. 2021). Taking into account our population sample, a low hepcidin status at baseline could be expected because of the aforementioned depleted ferritin stores and the fact that endurance training is suggested to present increased erythropoietic drive and iron needs (Pedlar et al. 2018). Therefore, if estradiol and progesterone had direct effects on resting hepcidin or on the hepcidin response to exercise, the aforementioned stimuli may mask the effects of sex hormones. This issue is a major concern when studying this population, as they often present with low iron stores and exercise-stressed erythropoiesis (Pedlar et al. 2018; Sim et al. 2019). Regardless, this study attempts to describe the hepcidin kinetics and iron homeostasis in this population as accurately as possible. For future research, it would be interesting to contrast our findings by performing a similar protocol in physically active women with repleted ferritin stores. Furthermore, although nutritional recommendations were provided to our participants and breakfast was replicated before testing, it would be worthwhile to carefully tailor energy and carbohydrate intake in relation to participants' fat-free mass in future studies. This may reduce the potential confounding effect of nutrition on the hepcidin response throughout the menstrual cycle (McKay et al. 2020). Finally, complementing our data, measuring additional time points between 3 and 24 h post-exercise; adding other variables related to erythropoiesis and hypoxia, such as circulating erythropoietin or erythroferrone; and quantification of iron losses through menstrual bleeding would be other issues to address to consider as many factors as possible that affect iron homeostasis in this population.

## Conclusion

This study describes, for the first time, the iron markers and hepcidin response to interval running exercise during the three most characteristic hormonal environments of the menstrual cycle, showing that the menstrual cycle phase significantly influence iron status in physically active eumenorrheic women, but the overall hepcidin response shows no clear changes and does not adjust concomitantly to these changes in iron status. However, it seems that in response to iron loss through menstruation, the hepcidin peak at 3 h after exercise may be slightly reduced in an attempt to maximize iron absorption during the EFP. These small changes in hepcidin seem to cater to the body's iron needs as a worsening of iron, ferritin and TSAT levels is found during EFP.

These results provide useful information to establish nutritional and supplementation strategies aimed at improving the availability of iron for iron-dependent body processes in physically active eumenorrheic women, suggesting that the EFP could present a greater iron absorption and, when needed, be the most suitable for supplementing with iron. Indeed, as the EFP showed reduced and limited serum iron and TSAT, boosting iron intake during this phase may help increase circulating iron levels to effectively supply erythropoiesis and prevent dysfunction of this important process. Finally, the variations observed in hepcidin and iron-related parameters within the menstrual cycle should be considered by clinicians in the diagnosis of ID and IDA in this population.

## Supplementary Information

Below is the link to the electronic supplementary material.

Supplementary file1 (DOCX 15 KB)

Supplementary file2 (DOCX 298 KB)
