# Correction to: Menstrual cycle affects iron homeostasis and hepcidin following interval running exercise in endurance-trained women

**DOI:** 10.1007/s00421-022-05064-5

**Published:** 2022-10-12

**Authors:** Víctor M. Alfaro-Magallanes, Laura Barba-Moreno, Nuria Romero-Parra, Beatriz Rael, Pedro J. Benito, Dorine W. Swinkels, Coby M. Laarakkers, Ángel E. Díaz, Ana B. Peinado, the IronFEMME Study Group

**Affiliations:** 1 LFE Research Group, Department of Health and Human Performance. Faculty of Physical Activity and Sport Science (INEF), Universidad Politécnica de Madrid, Martín Fierro, 7, 28040 Madrid, Spain; 2 Department of Laboratory Medicine, Translational Metabolic Laboratory (TML 830), Radboud University Medical Center, P.O. Box 9101, 6500 HB Nijmegen, The Netherlands; 3 Hepcidinanalysis.Com, Geert Grooteplein 10 (830), 6525 GA Nijmegen, The Netherlands; 4 Clinical Laboratory, National Center of Sport Medicine, Health and Sports Department, AEPSAD, Madrid, Spain

## Correction to: European Journal of Applied Physiology 10.1007/s00421-022-05048-5

The original version of this article unfortunately contained a mistake. The wrong figure appeared as Figs. 1 and 2. The Figs. 1 and 2 should have appeared as shown in the following page.

**Fig. 1:**
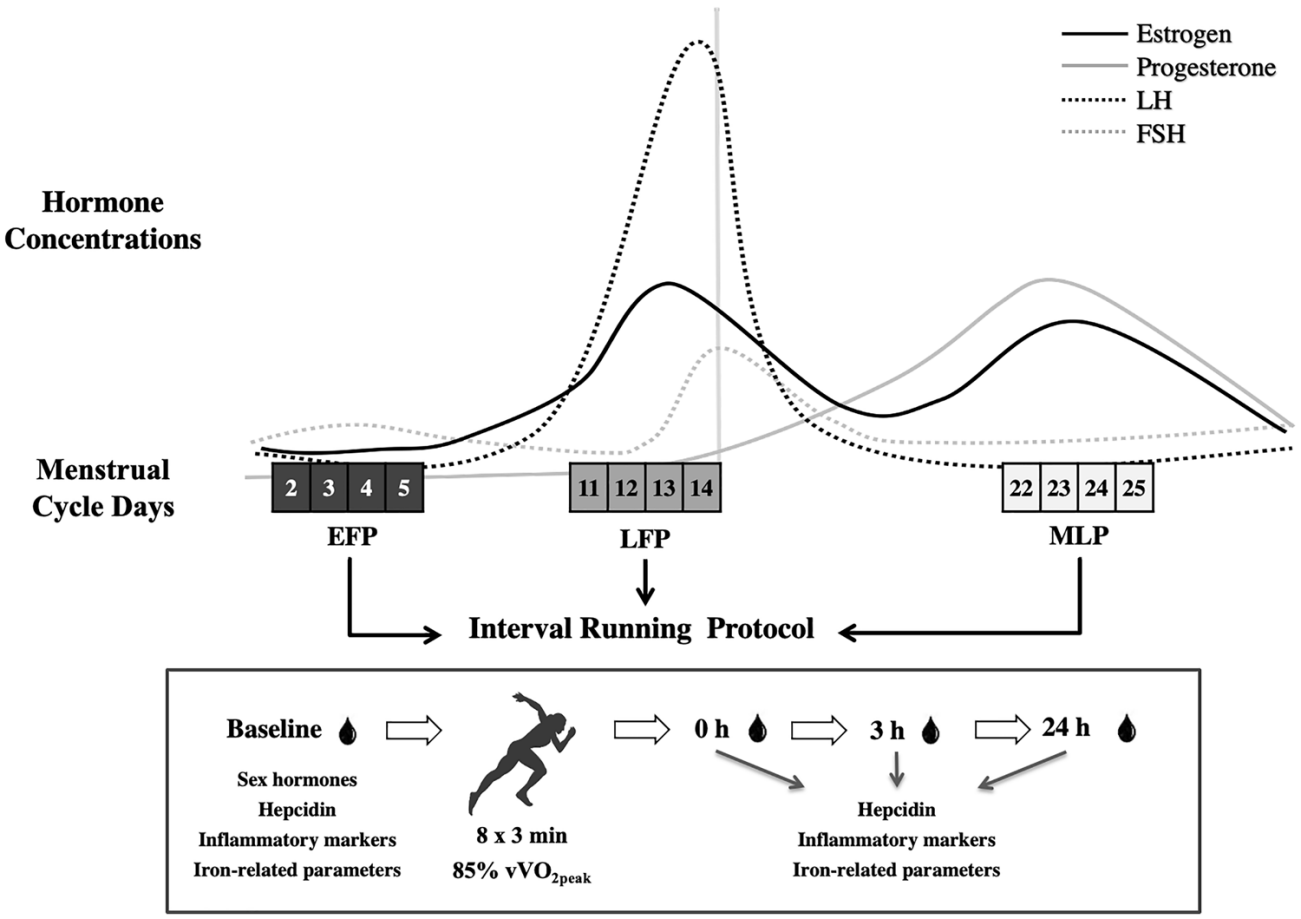
Experimental protocol performed in the EFP, LFP, and MLP of the menstrual cycle. *EFP* early-follicular phase, *FSH* follicle-stimulating hormone, *LFP* late-follicular phase, *LH* luteinizing hormone, *MLP* mid-luteal phase, 0 h, 0 h post-exercise, 3 h, 3 h post-exercise, 24 h, 24 h post-exercise

**Fig. 2:**
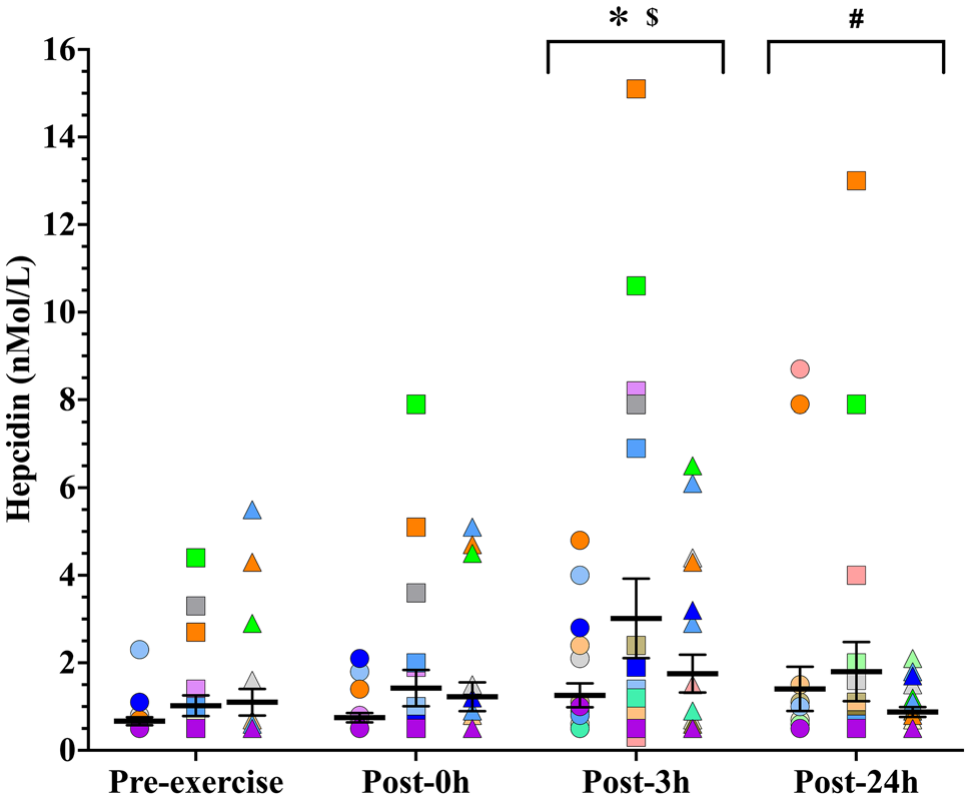
Mean (SEM) serum hepcidin concentrations and the individual hepcidin response of each participant to the interval running protocol in the early-follicular phase (circles), late-follicular phase (squares) and mid-luteal phase (triangles). Each color represents a different participant. Symbols above the arrows indicate post-hoc differences for the factor Time. Post-0 h, 0 h post-exercise; Post-3 h, 3 h post-exercise; Post-24 h, 24 h post-exercise *Significantly different from Pre-exercise. $ Significantly different from Post-0 h. # Significantly different from Post-3 h

