# Supplementary material for: Menstrual cycle affects iron homeostasis and hepcidin following interval running exercise in endurance-trained women
Source: Eur J Appl Physiol. 2022 Sep 21;122(12):2683–94. doi: 10.1007/s00421-022-05048-5 (PMC9613712; doi:10.1007/s00421-022-05048-5)
Supplement: Supplementary file 1 — Supplementary file1 (DOCX 15 KB) [file 421_2022_5048_MOESM1_ESM.docx]

**Supplementary Table 1**. Nutritional intake in each menstrual cycle phase during the test days presented as mean ± SD.

|  | **EFP** | **LFP** | **MLP** |
| --- | --- | --- | --- |
| **Energy intake (kcal)** | 1850.8 ± 543.9 | 1805.9 ± 426.4 | 1788.9 ± 539.0 |
| **Water (g)** | 2761.4 ± 696.3 | 2942.4 ± 667.5 | 2680.6 ± 720.1 |
| **Carbohydrate (g/kg)** | 2.4 ± 0.7 | 2.4 ± 0.9 | 2.2 ± 0.8 |
| **Protein (g/kg)** | 1.5 ± 0.5 | 1.6 ± 0.5 | 1.5 ± 0.5 |
| **Fat (g/kg)** | 1.8 ± 0.6 | 1.8 ± 0.6 | 1.8 ± 0.7 |
| **Iron (mg)** | 14.8 ± 3.8 | 14.9 ± 3.9 | 14.4 ± 3.4 |
| **Vitamin C (mg)** | 146.6 ± 73.3 | 177.2 ± 145.3 | 160.1 ± 78.9 |

EFP, early follicular phase; LFP, late follicular phase; MLP, mid-luteal phase.
