# Supplementary material for: Menstrual cycle affects iron homeostasis and hepcidin following interval running exercise in endurance-trained women
Source: Eur J Appl Physiol. 2022 Sep 21;122(12):2683–94. doi: 10.1007/s00421-022-05048-5 (PMC9613712; doi:10.1007/s00421-022-05048-5)
Supplement: Supplementary file 2 — Supplementary file2 (DOCX 298 KB) [file 421_2022_5048_MOESM2_ESM.docx]

**Supplementary Table 2** Time effects of the interval running protocol on hepcidin, inflammatory markers and iron-related parameters presented as mean (SEM).

|  | **Pre-exercise** | **Post-0h** | **Post-3h** | **Post-24h** |
| --- | --- | --- | --- | --- |
| **Hepcidin (nM)** | 0.93 ± 0.23 | 1.13 ± 0.31 | 2.01 ± 0.61 | 1.36 ± 0.49 |
| **Interleukin-6 (pg/ml)** | 1.70 ± 0.15 | 4.44 ± 0.49 | 1.71 ± 0.12 | 1.81 ± 0.23 |
| **TNF-⍺ (pg/ml)** | 4.55 ± 0.22 | 5.39 ± 0.37 | 4.83 ± 0.39 | 4.69 ± 0.21 |
| **CRP (mg/l)** | 0.91 ± 0.18 | 0.91 ± 0.19 | 0.86 ± 0.18 | 0.89 ± 0.14 |
| **Iron (µg/dl)** | 70.32 ± 7.83 | 76.29 ± 8.14 | 75.59 ± 7.46 | 80.35 ± 8.27 |
| **Ferritin (ng/ml)** | 36.39 ± 4.56 | 39.06 ± 4.82 | 37.67 ± 4.65 | 37.66 ± 4.75 |
| **Transferrin (mg/dl)** | 282.24 ± 6.73 | 297.11 ± 7.55 | 290.73 ± 7.80 | 283.37 ± 7.18 |
| **TSAT (%)** | 17.91 ± 2.02 | 18.60 ± 2.06 | 18.81 ± 1.92 | 20.42 ± 2.08 |

CRP, C-reactive protein; Post-0h, 0 hours post-exercise; Post-3h, 3 hours post-exercise; Post-24h, 24 hours post-exercise; TNF-⍺, tumor necrosis factor alpha; TSAT, transferrin saturation. * Significantly different from Pre-exercise. $ Significantly different from Post-0h. # Significantly different from Post-3h.
